# Comparative Studies of Cerebral Reperfusion Injury in the Posterior and Anterior Circulations After Mechanical Thrombectomy

**DOI:** 10.1007/s12975-021-00977-3

**Published:** 2022-01-10

**Authors:** Matthew M. Bower, Shuichi Suzuki, Kiarash Golshani, Li-Mei Lin, Mohammad Shafie, Hermelinda G. Abcede, Jay Shah, Dana Stradling, Wengui Yu

**Affiliations:** 1 Department of Neurology, University of California Irvine, 200 S. Manchester Ave., 206E, Orange, CA 92868 USA; 2 Department of Neurosurgery, University of California Irvine, 200 S. Manchester Ave., 206E, Orange, CA 92868 USA

**Keywords:** Cerebral reperfusion injury, Contrast extravasation, Posterior circulation, Symptomatic intracranial hemorrhage, Acute ischemic stroke, Mechanical thrombectomy

## Abstract

Cerebral reperfusion injury is the major complication of mechanical thrombectomy (MT) for acute ischemic stroke (AIS). Contrast extravasation (CE) and intracranial hemorrhage (ICH) are the key radiographical features of cerebral reperfusion injury. The aim of this study was to investigate CE and ICH after MT in the anterior and posterior circulation, and their effect on functional outcome. This is a retrospective study of all consecutive patients who were treated with MT for AIS at University of California Irvine Medical Center between January 1, 2014, and December 31, 2017. Patient characteristics, clinical features, procedural variables, contrast extravasation, ICH, and outcomes after MT were analyzed. A total of 131 patients with anterior circulation (AC) stroke and 25 patients with posterior circulation (PC) stroke underwent MT during the study period. There was no statistically significant difference in admission NIHSS score, blood pressure, rate of receiving intravenous tPA, procedural variables, contrast extravasation, and symptomatic ICH between the 2 groups. Patients with PC stroke had a similar rate of favorable outcome (mRS 0–2) but significantly higher mortality (40.0% vs. 10.7%, *p* < 0.01) than patients with AC stroke. Multivariate regression analysis identified initial NIHSS score (OR 1.1, CI 1.0–1.2, *p* = 0.01), number of passes with stent retriever (OR 2.1, CI 1.3–3.6, *p* < 0.01), and PC stroke (OR 9.3, CI 2.5–35.1, *p* < 0.01) as independent risk factors for death. There was no significant difference in functional outcomes between patients with and without evidence of cerebral reperfusion injury after MT. We demonstrated that AC and PC stroke had similar rates of cerebral reperfusion injury and favorable outcome after MT. Cerebral reperfusion injury is not a significant independent risk factor for poor functional outcome.

## Introduction

In 2015, 5 randomized controlled trials (RCTs) demonstrated the safety and efficacy of mechanical thrombectomy (MT) for patients with acute ischemic stroke (AIS) from large vessel occlusion (LVO) within 6–12 h of symptom onset [1–5]. In early 2018, 2 additional RCTs showed eloquent benefit of MT in select patients within 16 to 24 h of last known well [6, 7]. Of note, all of these RCTs were performed in patients with AIS in the anterior circulation (AC). Despite empirical evidence of potential benefit of MT for AIS in the posterior circulation (PC) [8–18], two recent clinical trials failed to prove the superiority of MT over standard medical therapy partly due to slow enrollment, small sample size, and inclusion of patients with minor deficit [19, 20].

Cerebral reperfusion injury is the major complication of MT. Contrast extravasation (CE) and intracranial hemorrhage (ICH) are the key radiographical features of cerebral reperfusion injury after MT [21–24]. In the setting of early ischemic change, the use of intravenous thrombolytic agent, mechanical endovascular thrombectomy device or large dose of contrast, and reperfusion may all potentially cause or worsen the blood–brain barrier (BBB) breakdown and cerebral reperfusion injury [23–26]. Both CE and ICH appear hyperdense on non-contrast CT scan. There is no threshold of Hounsfield units that can be reliably used to differentiate between blood and contrast. The iodinated contrast agents are generally washed out within 24 h [21, 22]. Therefore, a repeat CT scan within 24–36 h after MT is a useful tool in determining whether an initial CT hyperdense lesion represents CE or ICH. Persistent or new hyperdense lesion at 24–36-h follow-up CT is defined as ICH, whereas complete resolution comprises CE [22–24].

The aim of this study was to investigate CE and ICH after MT for PC and AC strokes and to examine if cerebral reperfusion injury affects outcome.

## Method and Materials

### Study Design

This retrospective study was approved by the Institutional Review Board at University of California, Irvine. The following clinical and radiographic information was collected from the electronic medical record and the Stroke Center’s American Heart Association (AHA) *Get with the Guidelines (GWTG)*-Stroke database: age, gender, race, past medical history, social or family history, the highest blood pressure (BP) levels within 24 h of admission, National Institutes of Health Stroke Scale (NIHSS) score before and after MT, noninvasive imaging or catheter angiography findings, the use of intravenous (iv) tPA, door to needle time (DNT), door to groin puncture time (DPT), procedural duration, number of passes, hyperdense lesion on CT scans, laboratory test results, and modified Rankin Scale (mRS) score at hospital discharge.

We evaluated whether there is difference in CE and ICH after MT between PC and AC stroke. The association between post-MT hyperdense lesions and functional outcomes was also examined.

### Patients

**Fig. 1:**
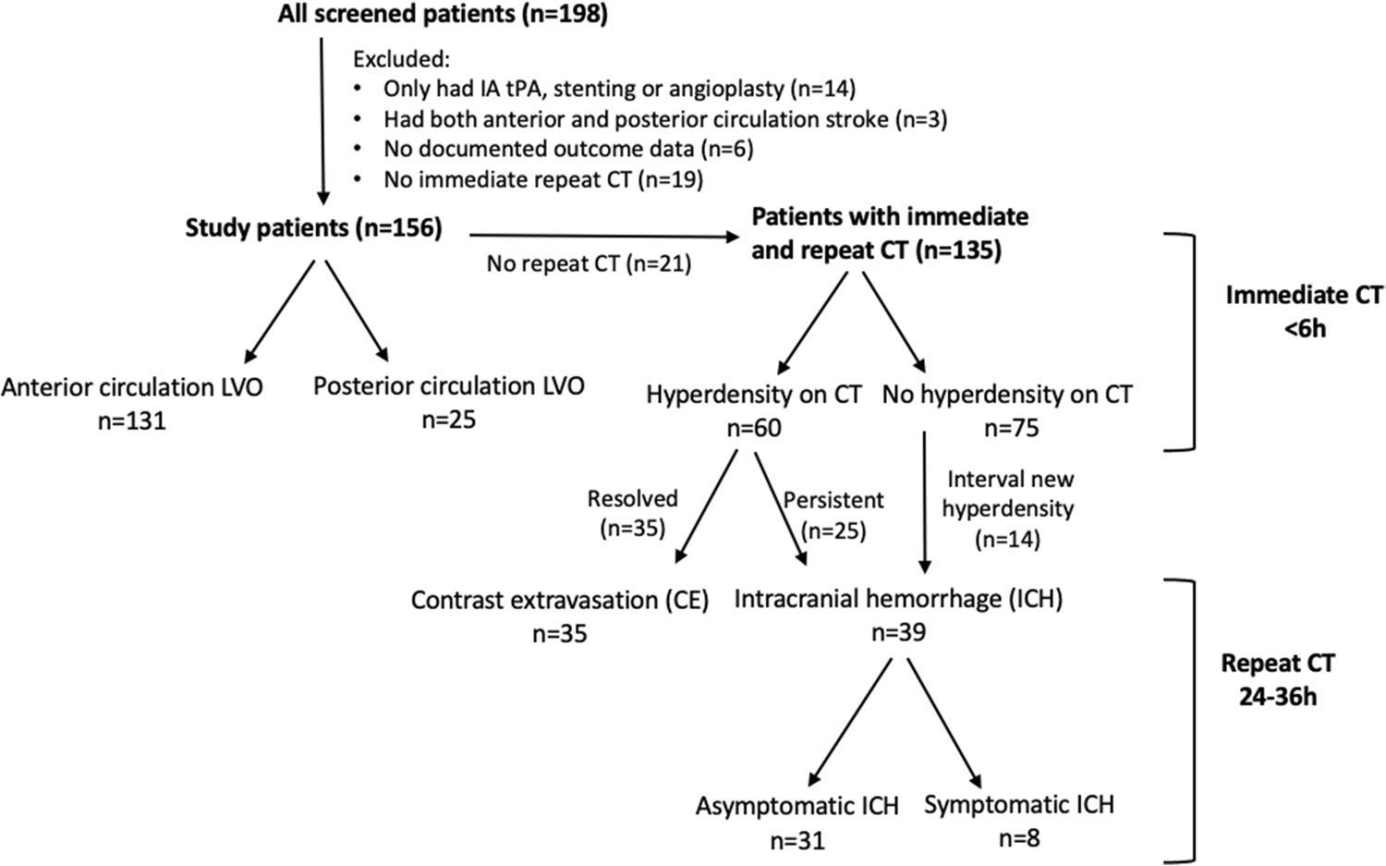
Study design and patient flowchart

All consecutive patients who underwent emergent MT for AIS in the anterior or posterior circulation at University of California Irvine Comprehensive Stroke Center between January 1, 2014, and December 31, 2017, were included. Patients were excluded if they had neither an immediate post-MT CT scan nor a 24–36-h follow-up CT or MRI. Patients were also excluded if there were AIS in both anterior and posterior circulations. The study flowchart is shown in Fig. 1.

### Protocol for MT

Our criteria for emergency MT were as follows: (1) patients presented within 6 h of symptom onset or wake up stroke; (2) NIHSS ≥ 6 and no evidence of hemorrhage on initial non-contrast CT scan; (3) no significant improvement after administration of intravenous tPA within 4.5 h of symptom onset; (4) evidence of large vessel occlusion in the anterior or posterior circulation on CTA; (5) ischemic penumbra ≥ one-third of the MCA territory on CT perfusion if patients presented with AIS in the anterior circulation 6–24 h of symptom onset or last known well. For PC stroke, MT was performed at the discretion of stroke and neuro-interventional team.

All MT procedures were performed under general anesthesia and using a stent retriever (Solitaire FR or Trevo), a direct contact aspiration device (Penumbra, Alameda, CA, USA), or both. After MT, the recanalization status was assessed using the Thrombolysis in Cerebral Infarction (TICI) scale [11]. Successful recanalization was defined as TICI 2 and TICI 3. Systolic BP was maintained at < 140 post-MT to minimize risk of reperfusion injury.

### Evaluation of CE and ICH

**Fig. 2:**
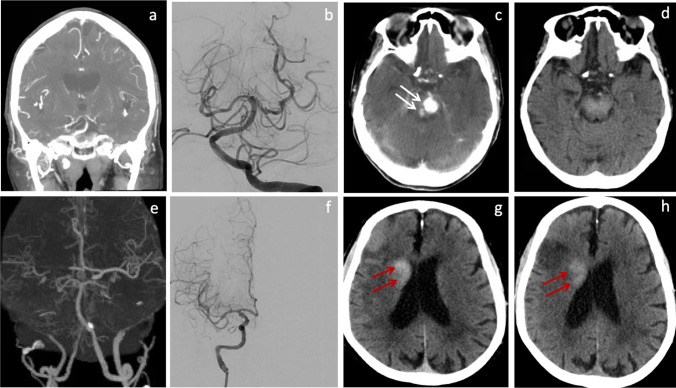
Upper panel: contrast extravasation (white arrow). A 75-year-old patient presented with drowsiness and speech difficulty. CTA showed distal BA occlusion (**a**). Immediately after endovascular recanalization (**b**), CT scan showed hyperdense lesions in the pons (**c**), which resolved on follow-up CT scan 24 h later (**d**). Lower panel: ICH (red arrows). A 74-year-old patient presented with slurred speech and left-sided weakness. CTA sowed right carotid terminus and MCA occlusion (**e**). Immediately after endovascular recanalization (**f**), CT scan showed hyperdense lesion in the right basal ganglia (**g**), which persisted on follow-up CT scan 24 h later (**h**)

Non-contrast CT scans were performed immediately and 24–36 h after MT in study patients. CE was defined as hyperdense lesion on the immediate post-MT CT but not on the 24–36-h follow-up scans. Persistent or new hyperdense lesion on 24–36-h follow-up CT was classified as ICH. In patients with follow-up MRI study, ICH was determined using susceptibility-weighted imaging (SWI). ICH includes intracerebral hemorrhage, subarachnoid hemorrhage (SAH), or subdural hemorrhage (SDH). Symptomatic ICH (sICH) was defined as evidence of ICH on CT scan and a neurologic deterioration with an increase in NIHSS score by more than 4 points [8]. Asymptomatic ICH (aICH) was defined as evidence of ICH on CT scan without significant neurologic deterioration. The representative images of the CE and ICH are shown in Fig. 2.

### Statistical Analysis

Continuous variables were described by mean ± standard deviation (SD) or median with interquartile range (IQR) based on the results of normality testing. Categorical variables were expressed by counts with percentages. The Student *t* test was used to analyze the difference in continuous variables between groups. The chi-square test or Fisher’s exact test was used in univariate analyses for categorical variables. Multivariate analysis was performed using multiple logistic regression analysis to evaluate factors that might predict functional outcome. All statistical analyses were done using SPSS version 23.0 (SPSS Inc., Chicago, IL, USA), and a *p* value less than 0.05 was considered statistically significant.

## Results

### Posterior vs Anterior Circulation Stroke

During the study period, a total of 198 patients underwent endovascular procedures for AIS from large vessel occlusion (Fig. 1). The following patients were excluded from the study: (1) patients received only intra-arterial tPA, stenting, or angioplasty (*n* = 14); (2) patients had stroke in both anterior and posterior circulation (*n* = 3); (3) patients had no documented outcome data (*n* = 6); and (4) patients had no CT scan within 6 h post-MT (*n* = 19).

Thus, 156 patients were included in the final analysis. Their baseline demographics and comorbidities are shown in Table 1. The mean ages were (70.1 ± 15.3). Of the 156 patients, 69 (44.2%) were males. There were 107 (68.6%) White/Hispanic, 36 (23.1%) Asian, 2 (1.3%) African American, 1 (0.6%) Native American/Pacific Islander, and 10 (6.4%) other ethnic groups. In total, 131 (84%) patients had AC stroke and 25 (16%) had PC stroke. The PC group had significantly higher rate of family history of stroke or TIA (36.0% vs 13.0%, *p* = 0.01) while the AC group had higher rates of atrial fibrillation/flutter (36.6% vs 12.0%, *p* = 0.02) and hyperlipidemia (42% vs. 20%, *p* = 0.04).

**Table 1:** Demographics and comorbidities in patients with anterior vs. posterior circulation stroke

	AC (*n* = 131)	PC (*n* = 25)	*p* value
Age (stdev)	71 (15.4)	67 (14.8)	0.24
Male, *n* (%)	58 (44.3)	11 (44.0)	0.98
African American, *n* (%)	2 (1.5)	0 (0.0)	0.54
White, *n* (%)	88 (67.2)	19 (76.0)	0.39
Asian, *n* (%)	31 (23.7)	5 (20.0)	0.69
Native American/Pacific Islander, *n* (%)	1 (0.8)	0 (0.0)	0.66
Other, *n* (%)	9 (6.9)	1 (4.0)	0.59
Past medical history			
HTN, *n* (%)	91 (69.5)	16 (64.0)	0.59
HLD, *n* (%)	55 (42.0)	5 (20.0)	**0.04**
A fib/A flutter, *n* (%)	48 (36.6)	3 (12.0)	**0.02**
DM, *n* (%)	42 (32.1)	4 (16.0)	0.11
CAD/MI/CHF, *n* (%)	47 (35.9)	10 (40.0)	0.7
Obesity, *n* (%)	25 (19.1)	2 (8.0)	0.18
OSA, *n* (%)	4 (3.1)	1 (4.0)	0.81
Stroke, *n* (%)	24 (18.3)	7 (28.0)	0.27
Carotid stenosis, *n* (%)	6 (4.6)	0 (0.0)	0.28
Prosthetic valve, *n* (%)	3 (2.3)	0 (0.0)	0.45
Drugs or alcohol use, *n* (%)	8 (6.1)	3 (12.0)	0.29
Smoking, *n* (%)	25 (19.1)	4 (16.0)	0.72
Fam history of stroke or TIA, *n* (%)	17 (13.0)	9 (36.0)	**0.01**

The clinical characteristics, procedural data, radiographical findings of cerebral brain injury, and functional outcome in patients with PC vs. AC stroke are shown in Table 2. There was no statistically significant difference between the 2 groups in admission NIHSS score, systolic BP, rate of receiving iv tPA, onset to puncture time, procedure time, passes, and recanalization rate. There was also no significant difference in the rates of hyperdense lesion on post-MT CT scan, contrast extravasation, or sICH. Both groups had a similar rate of favorable functional outcome (mRS 0–2) at 90 days (26.7% vs 20%, *p* = 0.1). However, PC stroke had a significantly higher mortality rate than the AC stroke (40.0% vs. 10.7%, *p* < 0.01).

**Table 2:** Admission characteristics, procedural factors, and functional outcome after MT in anterior vs. posterior circulation

	AC (*n* = 131)	PC (*n* = 25)	*p* value
Admission NIHSS (stdev)	16.8 (6.8)	17.0 (10.4)	0.93
Admission SBP (stdev)	159 (30.0)	172.6 (40.6)	0.11
tPA, *n* (%)	68 (51.9)	15 (60.0)	0.46
Onset to puncture time, min (stdev)	347.2 (238.2)	372.5 (215.8)	0.6
Procedure time, min (stdev)	69.5 (41.3)	81.9 (64.3)	0.36
Passes (stdev)	1.81 (1.16)	1.92 (1.0)	0.62
Recanalization (2b and 3), *n* (%)	118 (90.8)	21 (84)	0.31
Post-MT hyperdense lesion on CT	49 (37.4)	11 (44.0)	0.54
Contrast extravasation	30 (22.9)	5 (20.0)	0.75
sICH	6 (4.6)	2 (8.0)	0.48
mRS 0–2, *n* (%)	35 (26.7)	5 (20.0)	0.48
mRS 3–4, *n* (%)	49 (37.4)	5 (20.0)	0.1
mRS 5, *n* (%)	33 (25.2)	5 (20.0)	0.58
mRS 6, *n* (%)	14 (10.7)	10 (40.0)	** < 0.01**

**Fig. 3:**
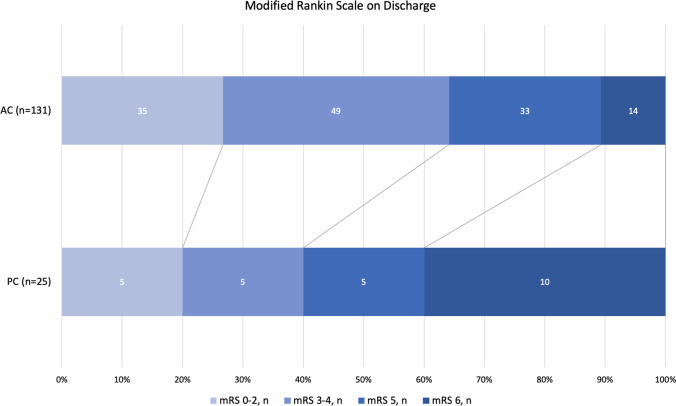
Functional outcomes of the anterior versus posterior circulation stroke after MT

The distribution of discharge modified Rankin Scale (mRS) is shown in Fig. 3. Multivariate regression analysis identified that the independent risk factors for death are initial NIHSS score (OR 1.1, CI 1.0–1.2, *p* = 0.01), number of passes with stent retriever (OR 2.1, CI 1.3–3.6, *p* < 0.01), and PC stroke (OR 9.3, CI 2.5–35.1, *p* < 0.01) (Table 3).

**Table 3:** Multivariate regression analysis of select variables and mortality

	OR	95% CI	*p* value
PC vs AC	9.4	2.5–35.1	** < 0.01**
Age	1	1.0–1.1	0.59
Initial NIHSS score	1.1	1.0–1.2	**0.01**
Male vs female	2	0.6–6.3	0.26
HLD vs no-HLD	1.8	0.6–5.6	0.29
AF vs no AF	0.7	0.2–2.5	0.62
tPA vs no-tPA	0.7	0.2–2.3	0.59
Onset to puncture time	1	1.0–1.0	0.32
Number of passes	2.1	1.3–3.6	** < 0.01**
Procedure duration	1	1.0–1.0	0.32

### Post-MT Hyperdense Lesion on CT Scan and Outcome

To investigate the possible effect of cerebral reperfusion injury on functional outcome, patients were divided into hyperdensity (HD) vs. no-hyperdensity (no-HD) group based on CT scan findings (Fig. 1). Among the 135 eligible patients, 60 had HD (44.4%) and 75 had no-HD on initial post-MT CT scan. Repeat CT scan within 24–36 h showed resolution of HD in 35 patients (CE group) and persistent HD in 25 patients (ICH group). There was new hyperdense lesion (ICH) in 14 patients who had no HD on initial CT scan, resulting in a total of 39 patients with ICH. Of the 39 patients with ICH, 8 were sICH (5.9%). There was no difference in sICH between PC and AC stroke (8% vs 4.6%) (Table 2). In one patient with basilar artery occlusion, the use of carotid angioplasty balloon caused dissection of atherosclerotic distal vertebral artery and SAH with intraventricular extension. The patient had extensive brainstem and bilateral occipital lobe infarcts likely due to basilar artery reocclusion.

As shown in Table 4, there was no significant difference between the HD and no-HD group in demographics, past medical history, social history of drug, alcohol, or tobacco use, as well as family history of stroke or TIA. There was also no significant difference in admission NIHSS score, SBP, procedural variables, and functional outcomes between the two groups (Table 5). There was a trend of higher rates of receiving iv tPA and favorable functional outcome in the no-HD group (Table 5). The rate of sICH was relatively low in both PC and AC (8% vs 4.6%). Radiographical evidence of cerebral reperfusion injury was not independently associated with poor outcome.

**Table 4:** Demographics and comorbidities in the post-procedural hyperdensity vs. no-hyperdensity groups

	HD (*n* = 60)	no-HD (*n* = 75)	*p* value
Age (stdev)	72 (13.5)	70 (15.6)	0.61
Male, *n* (%)	32 (53.3)	44 (58.7)	0.53
Af Am, *n* (%)	1 (1.7)	0 (0.0)	0.27
White, *n* (%)	40 (66.7)	52 (69.3)	0.74
Asian, *n* (%)	14 (23.3)	18 (24.0)	0.92
NA/PI, *n* (%)	1 (1.7)	0 (0.0)	0.27
Other, *n* (%)	4 (6.7)	5 (6.7)	1
Past medical history			
HTN, *n* (%)	44 (73.3)	50 (66.7)	0.41
HLD, *n* (%)	27 (45.0)	27 (36.0)	0.29
DM, *n* (%)	18 (30.0)	24 (32.0)	0.8
Afib/Aflutter, *n* (%)	24 (40.0)	21 (28.0)	0.15
Stroke, *n* (%)	14 (23.3)	14 (18.7)	0.52
Obesity, *n* (%)	11 (18.3)	13 (17.3)	0.88
CAD/MI/CHF, *n* (%)	22 (36.7)	24 (32.0)	0.57
Carotid stenosis, *n* (%)	1 (1.7)	4 (5.3)	0.27
OSA, *n* (%)	2 (3.3)	3 (4.0)	0.84
Prosthetic valve, *n* (%)	0 (0.0)	2 (2.7)	0.2
History of drugs/alcohol use, *n* (%)	6 (10.0)	4 (5.3)	0.31
Smoking, *n* (%)	10 (16.7)	17 (22.7)	0.39
Fam history of stroke or TIA, *n* (%)	9 (15.0)	14 (16.7)	0.57

**Table 5:** Admission characteristics, procedural factors, and functional outcome in post-MT hyperdensity (HD) vs. no-hyperdensity (no-HD) groups

	HD	no-HD	*p* value
Admission NIHSS score (stdev)	16.2 (7.4)	16.5 (7.4)	0.82
Admission SBP (stdev)	161.7 (29.2)	161.6 (30.7)	0.82
Anterior circulation, *n* (%)	49 (81.7)	66 (88)	0.3
IV tPA, *n* (%)	26 (43.3)	44 (58.7)	0.08
Onset to puncture time, min (stdev)	372.0 (221.0)	333.0 (228.0)	0.34
Passes (stdev)	1.92 (1.06)	1.74 (1.16)	0.35
Procedure time, min (stdev)	78.7 (53.8)	66.6 (40.9)	0.16
Recanalization (2b and 3), *n* (%)	53 (88.3)	67 (90.5)	0.68
mRS 0–2, *n* (%)	11 (18.3)	24 (32.0)	0.08
mRS 3–4, *n* (%)	20 (33.3)	27 (36.0)	0.74
mRS 5, *n* (%)	19 (31.7)	15 (20.0)	0.13
mRS 6, *n* (%)	10 (16.7)	9 (12.0)	0.45

In subgroup analysis, patients with contrast extravasation had a significantly higher rate of cardiac comorbidities compared to patients with ICH (51.4% and 23.1%, *p* = 0.016). The patients with sICH had a higher rate of diabetes than patients with aICH (75.0% vs. 30.3%, *p* = 0.015).

## Discussion

In this single center retrospective study, we demonstrated comparable rate of cerebral reperfusion injury and favorable outcome after MT in PC and AC. Patients with PC stroke had a higher rate of family history of stroke or TIA and mortality. In contrast, hyperlipidemia and atrial fibrillation were more common in patients with AC stroke. There was no statistically significant difference in hyperdense lesion, CE, ICH, and sICH after MT between PC and AC group. In multivariate regression analysis, PC stroke, initial NIHSS score, and number of passes were associated with higher mortality rate. Of note, there was no significant difference in functional outcome between the patients with and without reperfusion injury after MT. Cerebral reperfusion injury was therefore not a significant independent risk factor for mortality after MT.

Previous studies reported that prolonged procedure time increases periprocedural complications and is associated with increased rate of sICH [14, 27]. Raoult et al. found that a short onset to puncture time was associated with better outcomes [28]. Our study showed no correlation between procedural time and ICH or outcome, likely due to difference in sample size and patient selection. Both maximum blood pressure and large variations have also been shown to worsen outcome [29–31]. In our current study, there was no difference in initial SBP between the 2 groups and post-MT systolic BP was maintained at less than 140. Therefore, BP variation was unlikely the cause of higher mortality in patients with PC stroke. Of note, 85% of our PC stroke patients had acute basilar artery occlusion. This may be possible explanation for higher mortality rate in PC stroke as previous reported [15]. Other possible explanation can be re-occlusion of basilar artery or inclusion of patients with large pontine infarct [32], resulting in futile recanalization and possible higher rate of withdrawal of life support [15, 32]. The small sample size in the PC may be another confounding factor. Large vessel re-occlusion and futile recanalization after MT may be the next frontiers in translational stroke research.

Our data also demonstrated that there was no difference in the rate of good functional outcome (mRS 0–2) at hospital discharge between patients with and without hyperdense lesion on CT scan within 6 h of MT. Procedural variables such as onset to puncture time, procedural time, recanalization rate, and rate of receiving iv tPA were not significant predictors of development of hyperdense lesion. Our rate of hyperdense lesion (44.4%) is comparable to what were reported previously (30% and 55%) [23, 25]. Our rate of sICH (8%) in the PC stroke is also similar to what were reported in the 2 recently published RCTs (8% and 4.5%) [19, 20]. Since there was no difference in rate of hyperdense lesion, CE, or sICH between PC and AC group, indicating that cerebral reperfusion injury is not related to higher mortality rate in patients with PC stroke.

Of note, in subgroup analysis, we demonstrate that patients with CE had a significantly higher rate of cardiac comorbidities whereas the patients with sICH had a higher rate of diabetes. Pro-inflammatory cytokines and angiotensin II increases in heart failure were shown to downregulate the endothelial tight junctions of the blood–brain barrier, and this permeability increase has been measured with dynamic contrast-enhanced MRI [33]. Heart failure appeared to increase the risk of mild cerebral reperfusion injury (CE without ICH). In contrast, increased glucose levels were reported to increase cytotoxic edema and the risk for hemorrhagic transformation due to increase thrombotic and inflammatory response [34–37]. One study also reported the synergistic effect of high glucose and low BP on the risk for ICH [38]. Currently, it is unclear whether long standing diabetes, peri-procedural hyperglycemia, or both are responsible for increased risk of severe cerebral reperfusion injury.

The COMPASS trial assessed the efficacy of aspiration as first pass (*n* = 134) vs. stent retriever as first line (*n* = 136) [39]. It demonstrated no significant difference in outcome (52% vs. 50%) or intracranial hemorrhage (36 vs. 34%) between the 2 groups. We did not investigate the correlation between different thrombectomy device or combination and outcome. Instead, we examined the potential effect of the numbers of passes and procedure time on outcome and reperfusion injury. We found no significant correlation between the numbers of passes or procedure time and reperfusion injury after MT. However, multivariate regression analysis identified the number of passes as independent risk factor for death.

The major limitation of our study is the small sample size in the PC stroke group. The other limitation is that we do not have a dual energy CT, which was shown to improve accuracy in differentiating ICH from CE [22, 40].

Albeit not entirely accurate, regular CT is less resource-consuming and sensitive for the detection of CE and ICH [21, 24, 41–44]. Contrast agent was shown to be reabsorbed within 24 h and the persistence of hyperdensities after 19 to 24 h strongly favors ICH (specificity 100% and sensitivity 62.5%) [21]. MT induces abrupt reperfusion and increases the risk of BBB breakdown and secondary brain injury, predisposing patients to CE and ICH [41–44]. Regular CT is a very reliable tool for the evaluation of clinically relevant reperfusion injuries after MT.

## Conclusion

AC and PC stroke have similar rate of cerebral reperfusion injury and favorable outcome after MT. Cerebral reperfusion injury is not a significant independent risk factor for poor functional outcome.

## Data Availability

The data that support the findings of this study are available on request from the corresponding author.
