# Correction to: Comparative Studies of Cerebral Reperfusion Injury in the Posterior and Anterior Circulations After Mechanical Thrombectomy

**DOI:** 10.1007/s12975-022-00989-7

**Published:** 2022-02-17

**Authors:** Matthew M. Bower, Shuichi Suzuki, Kiarash Golshani, Li-Mei Lin, Mohammad Shafie, Hermelinda G. Abcede, Jay Shah, Dana Stradling, Wengui Yu

**Affiliations:** 1 Department of Neurology, University of California Irvine, 200 S. Manchester Ave., 206E, Orange, CA 92868 USA; 2 Department of Neurosurgery, University of California Irvine, 200 S. Manchester Ave., 206E, Orange, CA 92868 USA

**Correction to: Translational Stroke Research**

10.1007/s12975-021-00977-3

Figure caption for Figure 1 is incorrect. It contains submission discussion and only should include the first part “Study design and patient flowchart.”

The original article has been corrected.

